# Evaluation and Comparison of Academic Impact and Disruptive Innovation Level of Medical Journals: Bibliometric Analysis and Disruptive Evaluation

**DOI:** 10.2196/55121

**Published:** 2024-05-31

**Authors:** Yuyan Jiang, Xue-li Liu, Zixuan Zhang, Xinru Yang

**Affiliations:** 1 Henan Research Center for Science Journals Xinxiang Medical University Xinxiang China; 2 Faculty of Humanities & Social Sciences Xinxiang Medical University Xinxiang China

**Keywords:** medical journals, journal evaluation, innovative evaluation, journal disruption index, disruptive innovation, academic impact, peer review

## Abstract

**Background:**

As an important platform for researchers to present their academic findings, medical journals have a close relationship between their evaluation orientation and the value orientation of their published research results. However, the differences between the academic impact and level of disruptive innovation of medical journals have not been examined by any study yet.

**Objective:**

This study aims to compare the relationships and differences between the academic impact, disruptive innovation levels, and peer review results of medical journals and published research papers. We also analyzed the similarities and differences in the impact evaluations, disruptive innovations, and peer reviews for different types of medical research papers and the underlying reasons.

**Methods:**

The general and internal medicine Science Citation Index Expanded (SCIE) journals in 2018 were chosen as the study object to explore the differences in the academic impact and level of disruptive innovation of medical journals based on the OpenCitations Index of PubMed open PMID-to-PMID citations (POCI) and H1Connect databases, respectively, and we compared them with the results of peer review.

**Results:**

First, the correlation coefficients of the Journal Disruption Index (JDI) with the Journal Cumulative Citation for 5 years (JCC_5_), Journal Impact Factor (JIF), and Journal Citation Indicator (JCI) were 0.677, 0.585, and 0.621, respectively. The correlation coefficient of the absolute disruption index (Dz) with the Cumulative Citation for 5 years (CC_5_) was 0.635. However, the average difference in the disruptive innovation and academic influence rankings of journals reached 20 places (about 17.5%). The average difference in the disruptive innovation and influence rankings of research papers reached about 2700 places (about 17.7%). The differences reflect the essential difference between the two evaluation systems. Second, the top 7 journals selected based on JDI, JCC_5_, JIF, and JCI were the same, and all of them were H-journals. Although 8 (8/15, 53%), 96 (96/150, 64%), and 880 (880/1500, 58.67%) of the top 0.1%, top 1%, and top 10% papers selected based on Dz and CC_5_, respectively, were the same. Third, research papers with the “changes clinical practice” tag showed only moderate innovation (4.96) and impact (241.67) levels but had high levels of peer-reviewed recognition (6.00) and attention (2.83).

**Conclusions:**

The results of the study show that research evaluation based on innovative indicators is detached from the traditional impact evaluation system. The 3 evaluation systems (impact evaluation, disruptive innovation evaluation, and peer review) only have high consistency for authoritative journals and top papers. Neither a single impact indicator nor an innovative indicator can directly reflect the impact of medical research for clinical practice. How to establish an integrated, comprehensive, scientific, and reasonable journal evaluation system to improve the existing evaluation system of medical journals still needs further research.

## Introduction

Scientific and technical journals play a crucial role in showcasing research findings, and the value orientation of their published results is closely intertwined with their evaluation orientation. However, since Garfield [1] put forward the idea that “citation analysis can be used as an evaluation tool for journals” in 1972, the evaluation system of journals based on academic impact has become mainstream. However, relying too much on impact indicators for evaluation may hurt academic research and discipline progress.

On the one hand, some scholars have long pointed out that ranking journals according to their impact factors is noncomprehensive and may lead to misleading conclusions [2,3]. Meanwhile, many academic journals and publishers have engaged in strategic self-citation, leading to an overinflated journal impact factor (JIF) [4]. Some editorial behaviors to enhance the JIF clearly violate academic norms [5]. Some scholars are overciting each other’s work to enhance their academic impact [6]. External contingencies can have a devastating effect on citation indicators [7]. Scientists themselves also present a mixed attitude toward impact factors [8].

On the other hand, despite all the benefits of increased academic impact to journals, there is a nonnegligible problem in the evaluation of journals that citation indicators essentially characterize the impact of journals rather than their disruptive innovation [9]. Relevant studies have confirmed that the level of disruptive innovation of scientific research is getting increasingly lower [10] and the progress in various disciplines is slowing [11]. This is often overlooked against the background of impact-only evaluations. Therefore, despite the urgent need for disruptive innovations in science [12], impact-based journal rankings have made it more difficult to accept novel results [13], replacing the “taste of science” with the “taste of publication” [14] in the actual environment.

The evaluation of academic journals is about not only the journals themselves [15] but also the wide use of the evaluation results in academic review, promotion, and tenure decisions [16]. Meanwhile, the quality and results of medical research are directly related to human health and life and have a direct impact on human health and well-being. Therefore, the general and internal medicine journals indexed in Science Citation Index Expanded (SCIE) in 2018 were chosen as the study object. The OpenCitations Index of PubMed open PMID-to-PMID citations (POCI) and H1 Connect databases were selected as the sources for citation relationship data and peer review data. We investigated the connections and contrasts between the academic impact, disruptive innovation level, and results of peer review for medical journals and published research papers. We also analyzed the similarities and differences as well as the fundamental causes of the varying evaluation results in terms of impact evaluation, disruptive innovation, and peer review for various types of medical research papers. We aimed to provide a reference for the correct understanding of the innovation level of the results published by journals; the scientific and reasonable evaluation of medical journals; and the construction of a scientific, objective, and fair academic evaluation system.

## Methods

### Research Object

Because there is basically no disruptive innovation in the review literature, this paper only focuses on the general and internal medicine journals indexed in SCIE in 2018 and the research papers involved. In addition, considering the computational efficiency, accuracy, and difficulty of data acquisition, research papers for which citation relationships in the aforementioned journals were not included in POCI and the journals with too few references (less than 10) were excluded. Finally, 114 journals were retained at the journal level, and 15,206 research papers were retained at the paper level.

### Data Resource

The data acquired in this study included journal information, literature information, citation relationship data, and peer review data. The data were obtained through the Journal Citation Reports (JCR), Web of Science (WoS), POCI, and H1 Connect databases.

Of these databases, POCI is a Resource Description Framework (RDF) data set containing details of all the citations from publications bearing PMIDs to other PMID-identified publications harvested from the National Institutes of Health Open Citations Collection. POCI covers more than 29 million bibliographic resources and more than 717 million citation links (as of the last update in January 2023). Citations in POCI are not considered simple links but as data entities. This means that it is permissible to assign descriptive attributes to each citation, such as the date of creation of the citation, its time span, and its type [17].

H1 Connect (formerly Faculty Opinions), the world’s most authoritative peer review database in the biomedical field, incorporates the combined efforts of more than 8000 international authoritative experts from around the globe and is a knowledge discovery tool used to evaluate published research. H1 Connect’s reviewers are authoritative experts in the life sciences and medical fields. They provide commentary, opinions, and validation of key papers in their own field. The quality and rigor of the reviewers mean that researchers can be assured of the quality of the papers they recommend, and H1 Connect brings these recommendations together to recommend high-quality research to a wider audience. H1 Connect’s experts typically evaluate the “high level” of research literature in the field within 2 months of publication, with over 90% of recommendations made within 6 months of publication.

### Data Acquisition and Processing

The data acquired for this study consisted of 3 parts. The specific steps for data acquisition and processing were (1) data acquisition and (2) data processing.

#### Data Acquisition

The steps taken to acquire the data included the following: log in to JCR; select Medicine, General & Internal in the “Browse categories” page; select JCR Year=2018 and Citation Indexes=SCIE in the filters; export the result to XLS format; according to the acquired journal title, select the publication year as 2018 and the literature type as Article to search the WoS core database; and export the full record of the journal literature in XLS format. Finally, we downloaded the related H1 Connect literature data and POCI data according to the list of journals.

#### Data Processing

To process the data, we undertook the following steps: use Navicat to import the full record of research papers into the local SQLite database, process the downloaded POCI data, extract the PMID numbers of all the focus papers from the full record, retrieve the references and citations of the focus papers as well as the citations of the references of the focus papers in the local database transformed based on the POCI data, and establish the relevant data tables for the subsequent calculations.

### Evaluation Indicators

#### Innovation Indicators

Some researchers have observed, at an early stage, that some technological innovations complete and improve current technologies without replacing them while others outright eliminate technologies that were used in the past. However, for a long time, scholars did not analyze and explain the essence of this phenomenon. It was not until 1986 that Tushman and Anderson [18] summarized the phenomenon as follows: There are 2 types of major technological shifts that disrupt or enhance the capabilities of existing firms in an industry. However, Christensen [19], a professor at Harvard Business School in the United States, argued that disruptive innovations are new technologies that replace existing mainstream technologies in unexpected ways. Building on these views, Funk and Owen-Smith [20] provided deeper and more insightful insights. They argued that the dichotomy between disruptive and augmentative technologies lacks nuance and that the impact of new technologies on the status quo is a matter of degree rather than absolute impact.

In this regard, Govindarajan and Kopalle [21] also pointed out that disruptive innovation lacks reliable and valid measurement standards. Therefore, Funk and Owen-Smith [20] created the consolidation-disruption (CD) index, which aims to quantify the degree of technological change brought about by new patents. The index drew the attention of Wu et al [22], who analogized the basic principle of the CD index to measure disruption by calculating the citation substitution of the focus paper in the citation network and who were the first to apply the evaluation of disruptive innovation to the world of bibliometrics.

As an important carrier of academic results, it is important to evaluate papers quantitatively, rationally, and efficiently in terms of their innovation [23]. The disruption (D) index has received widespread attention after it was proposed by Wu et al [22]. A subset of scholars then explored disruptive papers in specific subject areas, including scientometrics [24], craniofacial surgery [25], pediatric surgery [26], synthetic biology [27], energy security [28], colorectal surgery [29], otolaryngology [30], military trauma [31], breast cancer [32], radiology [33], ophthalmology [34], plastic surgery [35], urology [36], and general surgery [37], based on the D index. Park et al [10] also analyzed the annual dynamics of the disruption level of papers and patents across the subject areas.

Another group of scholars conducted in-depth research on the index itself: Bornmann et al [38] explored the convergent validity of the index and the variants that may enhance the effectiveness of the measure and tested the validity of the D index based on the literature in the field of physics [39]. Ruan et al [40] provided an in-depth reflection on the limitations of the application of the D index as a measure of scientific and technological progress. Liu et al [41,42] empirically investigated the stabilization time window of the D index in different subject areas and addressed the mathematical inconsistency of the traditional D index and proposed an absolute disruption index (Dz; as in Equation 2) [43].

This series of studies has made it possible to evaluate the disruption of research papers based on the D index, which has gradually matured. On this basis, Jiang and Liu [44] proposed the Journal Disruption Index (JDI) to evaluate the disruptive innovation level of journals (as in Equation 3) and validated the evaluation effect of this indicator based on Chinese SCIE journals [45].



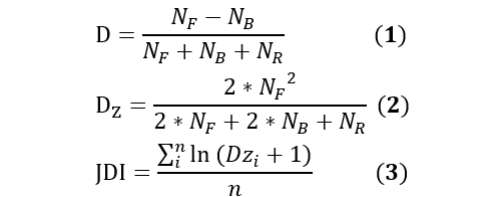



In Equations 1, 2, and 3, *N_F_* refers to the literature that only cites the focus paper (FP), *N_B_* refers to the literature that cites both the focus paper and at least one reference (R) of the focus paper, and *N_R_* refers to the literature that only cites at least one reference (R) of the focus paper but not the focus paper. n is the number of “Article” type pieces of literature contained in the journal, and Dz_i_ is the Dz of the i^th^ article in the journal.

In this study, Dz and JDI were chosen to evaluate the disruption of the selected studies at the literature and journal levels, respectively. Bornmann and Tekles [46] argued that 3 years is necessary regardless of the measurement for that discipline. Considering that, in the determination of the stabilization time window of the D index for each discipline conducted by Liu et al [42], the stabilization time window for clinical medicine is 4 years after publication. Therefore, in the process of calculating Dz in this paper, the citation time window of the focus papers was set to 2018 to 2022 to ensure the validity of the results. In addition, because the JDI value was too small, we multiplied the JDI by 1000 in all subsequent presentations.

#### Peer Review Indicators

The peer review indicators selected for this study included the peer review score (PScore), weighted peer review stars (PStar_w), and weighted peer review evaluation times (PTime_w). In this case, the weighted indicators refer to the weighting of ratings and number of evaluations by the number of evaluators when an evaluation was completed by more than one reviewer.

The advantage of using peer review indicators is that it can make up for the lag and one-sidedness of relying solely on the citations among the literature to assess the quality of the literature. It also corrects the shortcomings of the traditional JIF to judge the quality of the literature. Compared with a single impact indicator, it is more scientific.

#### Impact Indicators

The impact indicators selected for this study included the Cumulative Citation for 5 years (CC_5_), JIF, Journal Citation Indicator (JCI), and Journal Cumulative Citation for 5 years (JCC_5_). Among these, JIF is the total number of citations for scholarly articles published in the journal in the past 2 years divided by the total number of citable articles. JCI is the average Category Normalized Citation Impact (CNCI) of the citable literature published in the specific journal in the previous 3 years. The JCC_5_ is obtained by dividing the sum of the citation frequencies recorded in the POCI repository of the corresponding research papers (focus papers) of the selected journals for the years 2018 to 2022 by the number of research papers published by the journal (as in Equation 4).



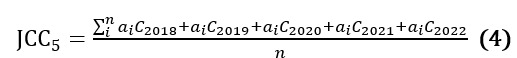



In Equation 4, a_i_C_t_ represents the number of citations of the i^th^ research paper of the journal in year t, and n is the number of research papers published by the journal.

## Results

### Evaluation and Correlation Analyses of Journals Under Different Evaluation Perspectives

#### Analysis of Differences in Academic Influence and Level of Disruptive Innovation of Journals

**Figure 1:**
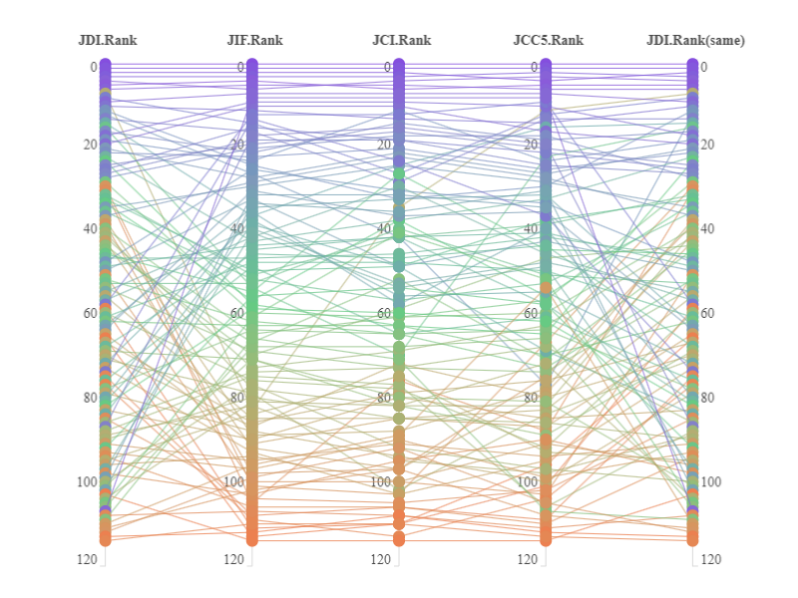
Comparison of ranking differences based on different evaluation indicators. JCC5: Journal Cumulative Citation for 5 years; JCI: Journal Citation Indicator; JDI: Journal Disruption Index; JIF: Journal Impact Factor.

The academic impact and level of disruptive innovation of the selected 114 journals, the results of the correlation analyses of the indicators, and the differences in the rankings are shown in Table 1, Table 2, and Figure 1, respectively. From these, we can see that (1) journals that are at the top of the ranking of impact indicators are usually also ranked at the top in the ranking of disruptive innovation, (2) journals at the bottom of the impact rankings are usually at the bottom of the innovation rankings, and (3) there is little difference in the ranking results of journals under different impact indicators, but there is a big difference in the ranking results of influence and disruptive innovation. Although there are moderate correlations between the JDI of journals and the 3 influence indicators of JIF, JCI, and JCC_5_, the average difference between the disruptive innovation ranking and academic influence ranking of the journals included in the study reached 20 places.

**Table 1:** Comparison of academic influence and disruptive innovation level of journals.

Journal	JCC_5_^a^	JDI^b^	JIF^c^	JCI^d^
*New England Journal of Medicine*	337.90	1167.64	70.67	24.83
*Lancet*	259.17	883.11	59.10	18.05
*Journal of the American Medical Association (JAMA)*	110.67	467.92	51.27	13.08
*Nature Reviews Disease Primers*	245.25	466.91	32.27	9.48
*Annals of Internal Medicine*	144.84	293.23	19.32	6.37
*British Medical Journal* *(BMJ)*	85.27	278.50	27.60	5.57
*JAMA Internal Medicine*	54.80	235.91	20.77	6.03
*International Journal of Evidence-Based Healthcare*	24.94	165.18	1.16	0.95
*Medical Journal of Australia*	24.67	162.99	5.44	1.18
*PLoS Medicine*	49.55	158.76	11.05	3.91
*Canadian Medical Association Journal*	23.64	138.67	6.94	2.06
*Journal of Travel Medicine*	12.63	136.54	4.16	0.98
*Journal of the Royal Society of Medicine*	6.00	121.29	3.54	0.83
*Amyloid*	24.28	105.39	4.92	1.13
*Medical Clinics of North America*	20.90	104.36	2.72	1.22
*Clinical Medicine*	17.59	101.48	2.05	0.52
*American Journal of Preventive Medicine*	19.70	95.50	4.44	1.74
*BMC Medicine*	28.06	82.00	8.29	2.74
*Annals of Family Medicine*	17.24	78.87	4.19	2.31
*Mayo Clinic Proceedings*	20.34	71.01	7.09	2.47
*American Journal of Chinese Medicine*	14.53	65.77	3.51	1.42
*British Journal of General Practice*	15.76	64.69	4.43	1.49
*Archives of Medical Science*	11.89	62.18	2.38	0.88
*Postgraduate Medical Journal*	7.54	61.38	1.98	0.61
*Journal of General Internal Medicine*	18.38	61.18	4.61	1.69
*Palliative Medicine*	17.67	60.71	4.96	1.56
*American Journal of Medicine*	16.70	55.79	4.76	1.56
*Deutsches Ärzteblatt International*	20.13	55.74	4.47	1.59
*Journal of the Chinese Medical Association*	8.10	53.26	1.89	0.62
*Journal of the National Medical Association*	6.91	48.41	0.83	0.24
*African Health Sciences*	5.25	44.92	0.78	0.44
*Journal of Urban Health*	11.17	42.83	2.15	0.83
*Canadian Family Physician*	11.27	42.82	2.19	0.77
*Family Practice*	8.13	41.90	1.99	0.88
*Journal of Pain and Symptom Management*	13.62	39.72	3.38	1.22
*Patient Preference and Adherence*	8.88	38.48	2.10	0.69
*Preventive Medicine*	13.94	37.36	3.45	1.35
*Saudi Medical Journal*	5.90	34.58	1.06	0.35
*Military Medicine*	6.43	34.01	0.85	0.28
*Journal of Korean Medical Science*	7.79	33.23	1.72	0.62
*Indian Journal of Medical Research*	5.59	32.76	1.25	0.39
*Sexual Medicine*	7.66	31.84	1.44	0.53
*Journal of the Royal Army Medical Corps*	5.90	31.27	0.99	0.28
*Journal of Evaluation in Clinical Practice*	10.02	30.32	1.54	0.64
*European Journal of General Practice*	6.68	29.86	1.62	0.76
*Journal of Women’s Health*	10.20	29.34	2.01	1.18
*International Journal of Medical Sciences*	10.61	29.30	2.33	0.81
*European Journal of Internal Medicine*	11.88	28.40	3.66	1.01
*BMJ Open*	11.00	28.20	2.38	0.83
*Journal of the Formosan Medical Association*	10.55	27.39	2.84	0.77
*Pakistan Journal of Medical Sciences*	3.64	26.87	0.83	0.27
*Medicine*	6.58	26.36	1.87	0.60
*Chinese Medical Journal, Peking*	9.08	25.93	2.10	0.85
*Scandinavian Journal of Primary Health*	6.49	25.93	1.56	0.49
*International Journal of Clinical Practice*	9.61	25.87	2.61	0.73
*Singapore Medical Journal*	5.55	25.68	1.14	0.37
*BMC Family Practice*	10.09	25.57	2.43	1.08
*Journal of Internal Medicine*	28.23	24.96	6.05	2.09
*Journal of the American Academy of Physician Associates*	3.91	24.76	0.46	0.14
*Journal of Postgraduate Medicine*	4.96	24.49	1.32	0.42
*Journal of Hospital Medicine*	9.25	24.46	2.28	0.79
*Internal Medicine Journal*	6.76	24.36	1.77	0.61
*Pain Medicine*	12.50	24.22	2.76	0.98
*Frontiers in Medicine (Lausanne)*	11.41	24.19	3.11	0.89
*Irish Journal of Medical Science*	5.26	23.52	1.03	0.37
*Acta Clinica Croatica*	4.28	23.25	0.40	0.14
*Clinics*	6.12	22.49	1.13	0.41
*Internal and Emergency Medicine*	10.29	21.32	2.34	0.80
*Medicina (Lithuania)*	5.25	21.31	1.47	0.39
*Medical Principles and Practice*	5.85	20.72	1.10	0.50
*Open Medicine (Warsaw, Poland)*	5.53	20.12	1.22	0.25
*Korean Journal of Internal Medicine*	8.34	19.75	2.71	0.70
*Colombia Médica*	4.33	17.23	0.98	0.24
*Journal of Clinical Medicine*	11.34	17.19	5.69	1.30
*Journal of Nippon Medical School*	2.39	17.11	0.62	0.19
*Postgraduate Medicine*	7.78	16.98	2.24	0.60
*British Journal of Hospital Medicine*	3.45	16.87	0.53	0.12
*Yonsei Medical Journal*	7.58	16.86	1.76	0.60
*São Paulo Medical Journal*	4.00	16.71	1.09	0.30
*Upsala Journal of Medical Sciences*	11.03	16.17	2.75	0.65
*Current Medical Research and Opinion*	8.00	15.73	2.35	0.83
*American Journal of the Medical Sciences*	7.71	15.52	1.96	0.63
*Revista Clínica Española*	5.34	15.19	1.04	0.40
*QJM: An International Journal of Medicine*	6.80	13.92	2.65	0.77
*Revista da Associacao Medica Brasileira*	3.78	13.73	0.80	0.23
*Libyan Journal of Medicine*	6.89	13.49	1.41	0.49
*Translational Research*	18.57	13.21	4.92	1.72
*Atención Primaria*	3.29	12.97	1.35	0.43
*La Presse Médicale*	4.16	12.95	1.17	0.29
*Balkan Medical Journal*	5.33	12.73	1.20	0.37
*Internal Medicine*	4.65	12.73	0.96	0.32
*Journal of Investigative Medicine*	8.00	12.54	1.99	0.71
*Southern Medical Journal*	4.12	12.42	0.87	0.33
*Scottish Medical Journal*	2.00	12.18	0.68	0.19
*World Journal of Clinical Cases*	4.74	12.01	1.15	0.45
*Acta Clinica Belgica*	4.98	11.76	0.96	0.27
*Diagnostics*	9.34	11.71	2.49	0.71
*Annals of Saudi Medicine*	5.22	11.08	0.81	0.29
*Annals of Medicine*	11.85	11.06	3.05	1.00
*Medicina Clinica (Barcelona)*	4.25	11.01	1.28	0.31
*Journal of Research in Medical Sciences*	5.63	10.95	1.47	0.40
*European Journal of Clinical Investigation*	12.57	10.76	2.78	0.92
*Journal of Nepal Medical Association*	1.27	10.23	0.21	0.07
*Wiener Klinische Wochenschrift*	6.66	10.10	1.17	0.39
*Journal of Medical Economics*	9.02	9.45	1.89	0.84
*Tohoku Journal of Experimental Medicine*	7.07	8.93	1.58	0.54
*Journal of Cachexia, Sarcopenia and Muscle*	41.24	6.90	10.75	3.08
*National Medical Journal of India*	1.68	5.92	0.64	0.18
*Croatian Medical Journal*	3.13	5.68	1.62	0.51
*Disease-a-Month*	8.44	5.13	0.94	0.34
*Revue de Médecine Interne*	2.97	5.02	0.81	0.28
*Medizinische Klinik, Intensivmedizin und Notfallmedizin*	3.34	3.52	0.85	0.31
*Internist*	1.58	2.67	0.43	0.18
*Hippokratia*	1.52	2.53	0.52	0.14

^a^JCC_5_: Journal Cumulative Citation for 5 years.

^b^JDI: Journal Disruption Index.

^c^JIF: Journal Impact Factor.

^d^JCI: Journal Citation Indicator.

**Table 2:** Correlation analysis of journal evaluation indicators.

Journal evaluation indicators		JDI^a^		JCC_5_^b^	JIF^c^		JCI^d^	
**JDI**								
	*r*		1	0.677		0.585		0.621
	*P* value		—^e^	<.001		<.001		<.001
**JCC_5_**								
	*r*		0.677	1		0.909		0.935
	*P* value		<.001	—		<.001		<.001
**JIF**								
	*r*		0.585	0.909		1		0.955
	*P* value		<.001	<.001		—		<.001
**JCI**								
	*r*		0.621	0.935		0.955		1
	*P* value		<.001	<.001		<.001		—

^a^JDI: Journal Disruption Index.

^b^JCC_5_: Journal Cumulative Citation for 5 years.

^c^JIF: Journal Impact Factor.

^d^JCI: Journal Citation Indicator.

^e^Not applicable.

#### Analysis of the Differences Between the Journals’ Impact and Disruption and the Results of Peer Review

In order to better analyze the differences between the academic impact and level of disruptive innovation with peer-reviewed results of journals in the field of general and internal medicine, papers indexed in H1 Connect were referred to as “H-papers,” and the source journals of “H-papers” are referred to as “H-journals.” The evaluation indicators, ranking of H-journals, and the percentage of H-papers are shown in Table 3 and Table 4. We can see that (1) the top 7 journals in terms of both academic impact and disruptive innovation are all H-journals, (2) the average impact ranking of H-journals is higher than the average innovation ranking, and (3) some journals with low impact and innovation also became H-journals.

**Table 3:** Evaluation indicators of H-journals (sources of papers indexed in the H1 Connect database).

Journal	Count, n	JPScore^a^	JPTime_w^b^	JFPStar_w^c^
*New England Journal of Medicine*	112	2192.2	234	528
*Lancet*	43	574.1	65	142
*Journal of the American Medical Association*	27	313.7	36	70
*Annals of Internal Medicine*	11	118.8	14	25
*Nature Reviews Disease Primers*	4	50.3	6	11
*British Medical Journal*	11	115.8	18	30
*Sexual Medicine*	3	14.1	7	7
*PLoS Medicine*	11	103.5	15	25
*JAMA Internal Medicine*	6	48.2	6	10
*International Journal of Clinical Practice*	3	23.2	3	5
*Journal of Cachexia, Sarcopenia and Muscle*	2	14	2	3
*American Journal of Medicine*	3	18.6	4	5
*BMC Medicine*	4	24.6	6	7
*Journal of Investigative Medicine*	1	4.7	1	4
*Medical Clinics of North America*	1	4.9	1	1
*Canadian Medical Association Journal*	1	9.4	1	2
*Medicina Clinica*	1	4.6	1	1
*Journal of Women’s Health*	2	9.5	2	2
*Wiener Klinische Wochenschrift*	1	4.6	1	1
*Mayo Clinic Proceedings*	1	4.6	1	1
*Frontiers in Medicine*	1	4.7	1	1
*Current Medical Research and Opinion*	1	4.7	1	1
*Pain Medicine*	1	4.7	1	1
*Preventive Medicine*	1	9.4	1	2
*BMJ Open*	5	28.3	5	6
*Journal of Clinical Medicine*	1	4.6	1	1
*Medicine*	5	28	5	6

^a^JPScore: journal peer review score.

^b^JPTime_w: weighted journal peer review evaluation time.

^c^JFPStar_w: weighted journal peer review star.

**Table 4:** Ranking and percentage of H-papers (papers indexed in the H1 Connect database) in H-journals (sources of H-papers).

Journal	JDI^a^ ranking	JIF^b^ ranking	JCI^c^ ranking	H-papers, n (%)
*New England Journal of Medicine*	1	1	1	112 (40.58)
*Lancet*	2	2	2	43 (22.51)
*Journal of the American Medical Association*	3	3	3	27 (18.24)
*Annals of Internal Medicine*	5	7	5	11 (11.46)
*Nature reviews. Disease primers*	4	4	4	4 (9.30)
*British Medical Journal*	6	5	7	11 (7.97)
*Sexual Medicine*	42	73	69	3 (7.14)
*PLoS Medicine*	10	8	8	11 (6.11)
*JAMA Internal Medicine*	7	6	6	6 (5.50)
*International Journal of Clinical Practice*	55	40	53	3 (4.62)
*Journal of Cachexia, Sarcopenia and Muscle*	107	9	9	2 (2.56)
*American Journal of Medicine*	27	19	19	3 (2.22)
*BMC Medicine*	18	10	10	4 (1.99)
*Journal of Investigative Medicine*	92	56	54	1 (1.52)
*Medical Clinics of North America*	15	37	25	1 (1.47)
*Canadian Medical Association Journal*	11	12	14	1 (1.28)
*Medicina Clinica*	100	77	92	1 (1.20)
*Journal of Women’s Health*	46	55	27	2 (1.19)
*Wiener Klinische Wochenschrift*	104	81	82	1 (1.10)
*Mayo Clinic Proceedings*	20	11	11	1 (0.94)
*Frontiers in Medicine*	64	31	37	1 (0.53)
*Current Medical Research and Opinion*	81	45	42	1 (0.50)
*Pain Medicine*	63	35	33	1 (0.48)
*Preventive Medicine*	37	29	23	1 (0.39)
*BMJ Open*	49	44	42	5 (0.24)
*Journal of Clinical Medicine*	74	14	24	1 (0.23)
*Medicine*	52	62	65	5 (0.16)

^a^JDI: Journal Disruption Index.

^b^JIF: Journal Impact Factor.

^c^JCI: Journal Citation Indicator.

### Evaluation and Correlation Analyses of Papers Under Different Evaluation Perspectives

#### Analysis of Differences in Academic Impact and Level of Disruptive Innovation of Papers

**Figure 2:**
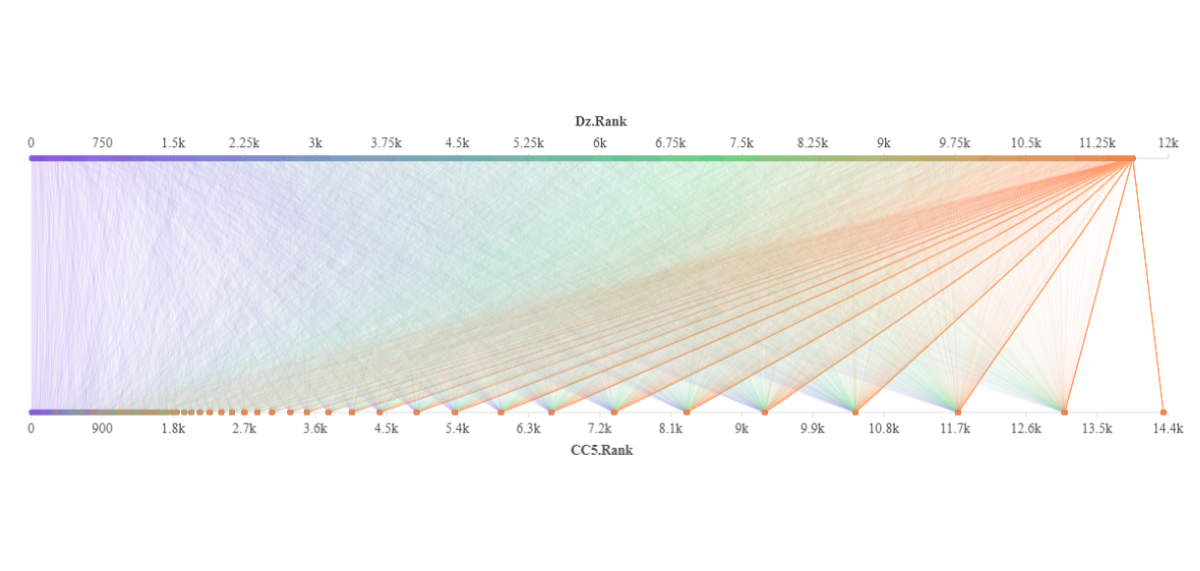
Comparison of the ranking of the absolute disruption index (Dz) and Cumulative Citation for 5 years (CC5) of selected research papers.

Ideally, if an article is accepted by a specific journal, it is because its overall quality is similar to other papers previously published in that journal [47]. However, journal-level indicators are, at best, only moderately suggestive of the quality of an article [48], which makes indicators that measure specific articles more popular [49]. Therefore, in this study, research papers in the field of general and internal medicine were also evaluated in terms of their academic impact and level of disruptive innovation, and the results are shown in Figure 2. From the results, we can see that research papers that rank high in the impact ranking usually also rank high in the disruptive innovation ranking. There were 8 (8/15, 53%), 96 (96/150, 64%), and 880 (880/1500, 58.67%) of the Top 0.1%, Top 1%, and Top 10% papers, respectively, selected based on the Dz and CC_5_ that were the same. Second, the level of disruptive innovation of research papers with the same level of impact varied greatly, and the impact level of research papers with the same level of disruptive innovation also varied greatly. Third, despite the high correlation (*r*=0.635, *P*<.001) between the Dz and CC_5_ of the selected research papers, the average difference between their innovation and impact rankings reached about 2700. Fourth, the actual analysis results showed no correlation between the innovation of the selected research paper and the number of references, which indicates that the Dz index is basically unaffected by the difference in the number of references in the actual evaluation process (*r*=0.006, *P*=.43).

#### Analysis of Differences Between Papers’ Impact and Disruption and the Results of Peer Review

**Figure 3:**
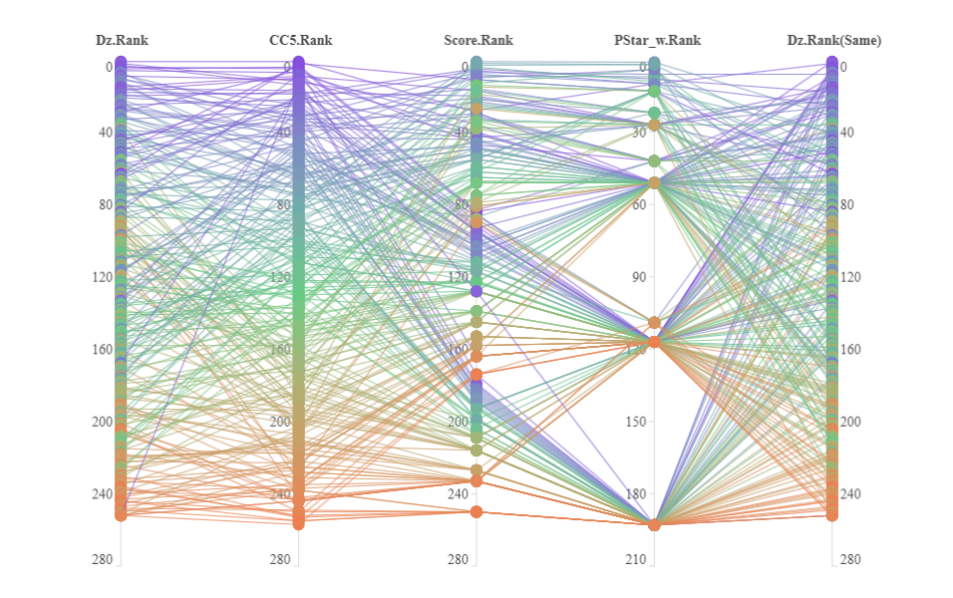
Comparison of ranking differences between H-papers (papers indexed in the H1 Connect database) in general and internal medicine based on different evaluation indicators. CC5: Cumulative Citation for 5 years; Dz: absolute disruption index; PStar: peer review stars.

In order to better analyze the differences between the academic impact, level of disruptive innovation, and peer review results of journal research papers in the field of general and internal medicine, the differences between the academic impact and disruptive innovation level and the peer review results of H-papers were analyzed. The specific results are shown in Table 5 and Figure 3. From the results, we see that there were 8 (8/15, 53%), 65 (65/150, 43.3%), and 187 (187/1500, 12.47%) H-papers among the top 0.1%, top 1%, and top 10% papers, respectively, selected based on Dz. There were 5 (5/15, 33%), 74 (74/150, 49.3%), and 220 (220/1500, 14.67%) H-papers among the top 0.1%, top 1%, and top 10% papers, respectively, selected based on CC_5_. Second, there was a significant positive correlation between the peer review indicators, disruptive innovation indicators, and academic impact indicators of H-papers, reflecting the consistency between the quantitative evaluation and peer review at the overall level. Third, the average impact ranking of H-papers was 865 (top 5.68%), and the average disruptive innovation ranking was 1726 (top 11.35%), which means that the average impact ranking of H-papers was higher than the average disruptive innovation ranking. Fourth, some papers with low academic impact and disruptive innovation level also became H-papers. Fifth, compared with the CC_5_, Dz has a minor correlation advantage with PTime_w and PStar_w.

**Table 5:** Validation of the correlation between 3 kinds of indicators of H-papers (papers indexed in the H1 Connect database).

Indicators		CC_5_^a^	Dz^b^	PTime_w^c^	PStar_w^d^	PScore^e^
**CC_5_**						
	*r*	1	0.749	0.292	0.385	0.613
	*P* value	—^f^	<.001	<.001	<.001	<.001
**Dz**						
	*r*		1	0.322	0.393	0.544
	*P* value	<.001	—	<.001	<.001	<.001
**PTime_w**						
	*r*	0.292	0.322	1	0.751	0.534
	*P* value	<.001	<.001	—	<.001	<.001
**PStar_w**						
	*r*	0.385	0.393	0.751	1	0.886
	*P* value	<.001	<.001	<.001	—	<.001
**PScore**						
	*r*	0.613	0.544	0.534	0.886	1
	*P* value	<.001	<.001	<.001	<.001	—

^a^CC_5_: Cumulative Citation for 5 years.

^b^Dz: absolute disruption index.

^c^PTime_w: weighted peer review evaluation times.

^d^PStar_w: weighted peer review stars.

^e^PScore: peer review score.

^f^Not applicable.

In addition to rating and commenting on the included research papers, different labels are added by the reviewers of articles in H1 Connect according to their different internal characteristics. The relevant definitions are shown in Table 6 (refer to Du et al [50]). We categorized and counted the 257 H-papers (in this study, if a paper had more than 1 tag, it was counted separately in the calculation of each tag), and the results are shown in Table 7. From this, we can see that (1) research papers with the tags in the “Novel Drug Target,” “Technical Advance,” and “New Finding” categories had a high academic impact and a high disruptive innovation level; (2) research papers with the “Changes Clinical Practice” tag showed only a moderate academic impact and disruptive innovation level but had high levels of peer-reviewed recognition and attention; (3) experts showed higher recognition and concern for research papers with tags of “Negative/Null Result,” “Controversial,” “Refutation,” and “Interesting Hypothesis,” but their academic impact and disruptive innovation level were lower than others.

**Table 6:** Categorization and definition of peer review tags.

Tag name	Definition
Confirmation	The article confirms previously published data or hypotheses.
Controversial	The article challenges established dogma.
Changes clinical practice	The article recommends that clinicians make complete, concrete and immediate changes in their practice.
Good for teaching	The article is a key article of significance in the field.
Interesting hypothesis	The article presents a meaningful model or hypothesis.
Negative/null result	The article has invalid or negative results
New finding	The article proposes a new data model or hypothesis.
Novel drug target	The article proposes a new drug target.
Refutation	The article refutes previously published data or hypotheses
Technical advance	The article introduces a new practical/theoretical technique, or a new use of an existing technique.

**Table 7:** Details of research papers with different peer-reviewed tags.

Type	Count, n	CC_5_^a^	Dz^b^	PScore^c^	PStar_w^d^	PTime_w^e^
Novel drug target	7	511.57	24.50	16.84	4.43	1.57
Technical advance	16	577.44	19.69	19.98	5.25	2.38
New finding	185	336.48	9.80	15.31	3.60	1.74
Confirmation	60	315.47	8.49	19.72	4.63	2.02
Good for teaching	82	248.77	7.47	18.12	4.63	2.22
Changes clinical practice	6	241.67	4.96	22.50	6.00	2.83
Negative/null result	27	281.81	3.84	17.81	4.52	2.30
Controversial	33	227.06	3.83	25.41	6.48	2.73
Refutation	4	167.75	2.94	22.53	6.00	3.25
Interesting hypothesis	37	207.86	1.77	20.68	5.38	2.24

^a^CC_5_: Cumulative Citation for 5 years.

^b^Dz: absolute disruption index.

^c^PScore: peer review score.

^d^PStar_w: weighted peer review stars.

^e^PTime_w: weighted peer review evaluation times.

## Discussion

### Principal Findings

#### Evaluation of Disruptive Innovation Is Detached From the Traditional Evaluation System

From these research results, large differences were seen between the innovation and impact rankings of the journals and research papers included in the study. This is also consistent with the findings of Guo and Zhou [51]. This phenomenon reflects the essential difference between the 2 different evaluation systems. It also proves that the evaluation of the disruptive innovation of research papers and journals based on Dz and JDI is not consistent with the traditional evaluation system of impact.

The essence of disruptive evaluation is to measure the innovation from the substitution level of knowledge structure. This evaluation method brings new ideas to the field of scientific research evaluation, helps relevant institutions and scholars remove the constraints of the traditional evaluation system, and helps to establish a value orientation of encouraging innovation for scientific research and scientific and technological journals, so as to promote the benign development of the academic ecology.

#### The 3 Evaluation Systems Only Have High Consensus on the Top Object

Although, given the consistency of scientific evaluation, there will be some uniformity in the level of disruptive innovation, level of academic impact, and peer review results of journals as well as research papers.

However, from the research results presented in this paper, we can see that (1) the top 7 journals in terms of both academic impact and disruptive innovation were all H-journals, (2) more than one-half of the top papers selected based on Dz and CC_5_ were the same, (3) the average H-papers were ranked at the top in terms of impact and innovation, and (4) the results of the different evaluation systems only had high consensus on the authoritative journals and top papers in the field.

These findings are also consistent with those of Goman [52] and Chi et al [53]. A fundamental reason for this phenomenon is that the purposes of the 3 evaluation systems are inherently different. Therefore, in the actual evaluation process, the 3 kinds of indicators are not interchangeable, and the combination of the 3 evaluation systems may be a feasible solution to establish a comprehensive, scientific, and reasonable journal evaluation system.

#### A Single Indicator Cannot Accurately Reflect the Impact of Medical Research on Clinical Practice Alone

From the aforementioned findings, we can see that research papers of the “Novel Drug Target,” *“*Technical Advance,” and “New Finding” types have high academic impact and high levels of innovation, which is in line with their classification definitions. This is also consistent with the findings of Du et al [50], Thelwall et al [54], and Jiang and Liu [55]. Second, research papers of the “Changes Clinical Practice” type showed only moderate levels of innovation and impact but had high levels of peer-reviewed recognition and attention. This reflects the difficulty of evaluating the impact of a particular academic paper on clinical practice, whether the evaluation system is based on academic impact or level of disruptive innovation. Third, peer-reviewed experts show higher recognition and concern for research papers of the types “Negative/Null Result,” “Controversial,” “Refutation,” and “Interesting Hypothesis,” but the level of impact and disruptive innovation of these papers are lower. This partly reflects the current academic community’s excessive focus on positive results; deliberate avoidance of negative results; and overall lack of support for debatable, falsified, and other types of research.

Several scholars have recently suggested that the evaluation system of medical journals should be redesigned for contemporary clinical impact and utility [56]. In this regard, Thelwall and Maflahi [57] advocated that the references of a guideline are an important basis for studying clinical value. However, Traylor and Herrmann-Lingen [58] found only a weak correlation between the number of citations to individual journals in the guidelines and their respective JIF. Therefore, the JIF is not a suitable tool for assessing the clinical relevance of medical research. The results of this study similarly found that research papers of the “Changes Clinical Practice” type showed only moderate levels of disruptive innovation and academic impact, but such research papers received higher recognition and attention from peer review experts. Therefore, combining quantitative evaluation with peer review may be a feasible way to measure the impact of medical research on clinical practice.

### Limitations

However, this study also has the following limitations. First, papers in the medical field have a preference for citing review articles [59], which has a certain impact on the evaluation of the disruptive innovation of research papers. Second, the scoring mechanism provided to its reviewers by H1 Connect has a low differentiation degree and cannot perfectly distinguish the differences in quality between papers yet. In addition, H1 Connect has too few evaluators. Brezis and Birukou [60] illustrated that, if the number of reviewers is increased to about 10, the correlation between the results and the quality of the paper will be significantly improved. However, it is difficult to seek so many high-quality experts who are willing to accept open peer review in the high pressure environment of “Publish or Perish” [61]. Third, since the citation data sources used in this study are all based on PubMed data, this study also suffers from the problem of missing references and citations that are not labeled with a PMID, which affects the accuracy of the evaluation results to a certain extent. In future studies, we will obtain more accurate measurement results by jointly using multiple sources of citation data.

### Conclusions

In this study, the general and internal medicine journals indexed in SCIE in 2018 were chosen as the study object. The POCI and H1 Connect databases were selected as sources of citation relationship data and peer review data. We investigated the connections and contrasts between the academic impact, level of disruptive innovation, and results of peer review for medical journals and published research papers. We also analyzed the similarities and differences as well as the fundamental causes of the varying evaluation results in terms of impact evaluation, disruptive innovation, and peer review for various types of medical research papers.

The results of this study show that the evaluation of scientific research based on the innovation index is detached from the traditional impact evaluation system, the 3 evaluation systems only have high consistency for authoritative journals and top papers, and neither the single impact index nor the innovation index can directly reflect the impact of medical research on clinical practice.

In addition, with the increasing importance of replicative science, the accuracy of statistical reports, evidential value of reported data, and replicability of given experimental results [62] should also be included in the examination of journal quality. How to establish a comprehensive, all-encompassing, scientific, and reasonable journal evaluation system needs to be further investigated.

## Abbreviations

CC5: Cumulative Citation for 5 years
CD: consolidation-disruption
CNCI: Category Normalized Citation Impact
D: disruption
Dz: absolute disruption index
JCC5: Journal Cumulative Citation for 5 years
JCI: Journal Citation Indicator
JCR: Journal Citation Reports
JDI: Journal Disruption Index
JIF: Journal Impact Factor
POCI: the OpenCitations Index of PubMed open PMID-to-PMID citations
PScore: peer review score
PStar_w: weighted peer review star
PTime_w: weighted peer review time
RDF: Resource Description Framework
SCIE: Science Citation Index Expanded
WoS: Web of Science

## Data Availability

All citation data can be obtained from [63].
